# MiR-642a-3p increases the risk of postmenopausal osteoporosis and fractures by targeting INO80

**DOI:** 10.1186/s41065-026-00689-4

**Published:** 2026-05-13

**Authors:** Jingyu Zhang, Han Xiao

**Affiliations:** Department of Traumatic Orthopedic, The First Affiliated Hospital of Harbin Medical University, No. 23, Postal Street, Nangang District, Harbin, 150000 China

**Keywords:** MiR-642a-3p, INO80, Postmenopausal osteoporosis, Fractures, Osteoblast

## Abstract

**Background:**

Postmenopausal osteoporosis (PMO), a metabolic bone disorder from estrogen deficiency, disrupts the balance between bone resorption and formation. Its severe consequence, osteoporotic fractures (OPF), significantly increases disability and mortality, severely impacting patients’ quality of life. Elevated miR-642a-3p has been identified in both PMO and bone loss. This research aims to further explore the role of miR-642a-3p in predicting PMO development and OPF risk, along with its underlying mechanisms.

**Results:**

The research enrolled 127 PMO patients and 91 healthy volunteers (HV). And further subdivided the PMO group into osteoporotic fractures (*n* = 62) subgroup and without fractures (OPNF, *n* = 65) subgroup. The results indicate that miR-642a-3p was significantly upregulated in both the PMO and OPF groups. MiR-642a-3p demonstrated good predictive performance for PMO occurrence and OPF risk. High miR-642a-3p expression was identified as an independent risk factor for progression from PMO to OPF. Downregulation of miR-642a-3p not only stimulated osteoblast proliferation but also mitigated associated inflammation and oxidative stress. Furthermore, INO80 is targeted and regulated by miR-642a-3p.

**Conclusion:**

MiR-642a-3p may have potential as a biomarker for future clinical prediction of PMO and OPF. Inhibition of miR-642a-3p promotes osteoblast proliferation, associated inflammation, and oxidative stress by upregulation of INO80 expression.

## Background

Postmenopausal osteoporosis (PMO) is a systemic skeletal disorder characterized by reduced bone mass, compromised bone microarchitecture, and diminished bone tissue strength [1]. And its primary pathogenesis is believed to stem from disrupted bone remodeling equilibrium caused by declining levels of various sex hormones, including postmenopausal estrogen (E2) [2]. Furthermore, osteoporosis increases skeletal fragility, leading to fractures. Globally, osteoporosis causes approximately 9 million fractures annually [3]. Osteoporotic fractures (OPF) most frequently occur in the vertebrae, hip, and wrist. Hip fractures carry a significant burden of morbidity and mortality, with a first-year mortality rate of about 20% and a recurrence rate of approximately 6.07% [4]. The PMO presents with no obvious clinical symptoms in its early stages; it is often only diagnosed after adverse events such as bone pain or fractures [5]. Therefore, early prediction and treatment of fractures are critically important.

Along with estrogen deficiency, inflammatory responses, oxidative stress, and dysregulated microRNAs (miRNAs) expression are also recognized as contributing factors to bone loss and PMO [6]. MiRNAs are key regulators in the progression of PMO and OPF by modulating the proliferation, differentiation, and apoptosis of various bone cells, such as osteoblasts, osteoclasts, and osteocytes [7, 8]. Research has confirmed that miR-642a-3p is linked to the malignant progression of various diseases, such as diabetes [9], hypertrophic cardiomyopathy [10], and multiple cancers (gallbladder cancer and hepatocellular carcinoma) [11, 12]. The previous study has reported that miR-642a-3p is significantly upregulated in both PMO and bone loss by comparing miRNA expression profiles [13]. Consequently, we hypothesized that miR-642a-3p is associated with PMO and OPF. The role and underlying mechanisms of miR-642a-3p were further investigated in this study.

MiRNAs mediate their regulatory effects by binding to the 3’-UTR of target mRNAs, thereby influencing the development of osteoporosis and fracture healing [14, 15]. Therefore, studying the interaction between miRNAs and mRNA will aid in the elucidation of the intrinsic mechanisms for miR-642a-3p functional differentiation. As a chromatin remodeling complex, INO80 is closely implicated in various bone-related diseases such as osteoporosis, osteoarthritis, and osteosarcoma through its multifaceted regulation of key osteoblast processes, including stem cell properties, differentiation, proliferation, senescence, and apoptosis [16, 17]. And INO80 effectively repairs DNA damage accumulated by osteoblasts throughout their lifecycle, preventing damage accumulation and osteoblast senescence [18]. In the study, INO80 is a target gene of miR-642a-3p according to the TargetScan database. The potential association between miR-642a-3p and INO80 warrants further investigation.

The conventional diagnostic criterion for osteoporosis is a BMD T-score of less than − 2.5, as measured by dual-energy X-ray absorptiometry [19]. And the fracture risk assessment tool is designed to estimate an individual’s probability of having a fracture [20]. However, these methods cannot accurately identify osteoporosis patients at high fracture risk to guide treatment, nor can they continuously monitor fracture risk and clinical efficacy post-treatment [21]. Therefore, it is crucial to the ongoing exploration of novel and efficient biomarkers. This study aims to investigate the role of miR-642a-3p in PMO and OPF and its potential molecular mechanisms, which will facilitate earlier diagnosis and therapeutic evaluation of PMO and OPF.

## Materials and methods

### Clinical samples

Blood samples were collected from 91 healthy volunteers (HV) and 127 PMO patients from July 2021 to July 2022. Based on the occurrence of fragility fractures, 127 PMO patients were further categorized into 62 with OPF and 65 without fractures (OPNF). The study protocol was approved by the Ethics Committee of The First Affiliated Hospital of Harbin Medical University (No. 2021-0047), and informed consent was obtained from all participants. All procedures were performed in line with the principles of the Declaration of Helsinki. Inclusion criteria are as follows: (1) PMO patients meeting diagnostic criteria for osteoporosis, Bone Mineral Density (BMD) T-score <−2.5 standard deviations; (2) OPF patients with newly diagnosed fractures confirmed by imaging (within 2 weeks of onset). Exclusion criteria included: (1) use of corticosteroids. (2) Fractures resulting from high-energy trauma or non-osteoporotic pathologies; (3) Presence of diseases affecting bone metabolism (e.g., malignancy, hyperparathyroidism) or autoimmune disorders (rheumatoid arthritis); (4) chronic liver disease (5) Kidney failure or disease (6) Use of anti-osteoporosis medications (e.g., bisphosphonates, calcitonin) within the past 3 months. Meanwhile, 91 healthy elderly women with normal BMD, who received physical examinations concurrently, were enrolled as the control group. They were matched with the PMO patient group in terms of age and BMI, with normal BMD.

### Cell culture and transfection

The MC3T3-E1 cells (ATCC, Manassas, VA, USA), characterized by rapid proliferation, stability, and ease of culture, serve as a representative in vitro model for bone metabolism research. The MC3T3-E1 cellswere cultured in Minimum Essential Media – Alpha (alpha-MEM; Hyclone, Cytiva, Marlborough, MA, USA) containing 10% Fetal Bovine Serum (FBS, Gibco), 1% penicillin/streptomycin at 37℃ with 5% CO_2_. MiR-642a-3p inhibitor (GGUUCCCUCUCCAAAUGUGUCU) and inhibitor-NC (AAGGCUAGCAUAGAAUCGUA) were purchased from RiboBio (Guangzhou, China) and transfected into MC3T3-E1 cells via Lipofectamine 2000 (Thermo Fisher Scientific, Inc.). Briefly, 100 pmol of miR-642a-3p inhibitor (inhibitor-NC) was diluted in 250 µL of Opti-MEM medium (Gibco), and 5 µL of Lipofectamine 2000 was diluted in another 250 µL of Opti-MEM. The two solutions were mixed gently (1:1 ratio) and incubated for 20 min at room temperature to form transfection complexes. The mixture (500 µL) was then added to each well containing 1.5 mL of Opti-MEM, resulting in a final inhibitor concentration of 50 nM. After 6 h of incubation, the medium was replaced with fresh alpha-MEM containing 10% FBS. The transfection efficiency was confirmed by Osteogenic differentiation was induced using 50 mg/L ascorbic acid (AA) and 10 mM/L β-glycerophosphate (β-GP). After 15 days of differentiation, expression of miR-642a-3p, INO80, and osteoblast differentiation markers was detected via RT-qPCR.

### Extraction of total RNA and RT-qPCR

The blood samples were collected into EDTA anticoagulant tubes, and plasma was separated by centrifugation at 2500 g for 15 minutes at 4°C. The supernatant was collected after centrifuging again and stored at −80°C. cDNA was synthesized utilizing the RevertAid™ H Minus First Strand cDNA Synthesis Kit (Fermentas; Hanover, NH, USA). RT-qPCR was conducted employing the ABI 7500 Real-Time PCR System (Biosystems; Foster, CA, USA). miR-642a-3p, 5’-AGACACATTTGGAGAGGGAACC-3’ (forward), and the downstream primer is the universal primer supplied with the kit. INO80, 5’-CGGAATCGGCTTTTGCTA-3’ (forward) and 5’-TGTCGGCTGGTCAGTTGG-3’ (reverse). Next, U6 (forward 5’-CTCGCTTCGGCAGCACA-3’ and reverse 5’-AACGCTTCACGAATTTGCGT-3’) and GAPDH (forward 5’-ACTGGCATGGCCTTCCGT-3’ and reverse 5’-CCACCCTGTTGCTGTAGCC-3’) were used as controls for miR-642a-3p and INO80. And their expressions were quantified through the 2^−∆∆ct^ method.

### CCK8 assay

Cells in the logarithmic growth phase (1 × 10⁴) were trypsinized and resuspended in culture medium, then seeded into a 96-well plate, and incubated for 24, 48, or 72 h. Then, 10 µL CCK-8 (Dojindo, Japan) was added to each well. Subsequently, the plates were incubated at 37^◦^C for 2 h. Cell viability was measured using a CCK-8 assay kit (Beyotime, Shanghai, China), and the OD at 450 nm was detected with a microplate reader (BMG LABTECH, Offenburg, Germany).

### Detection of inflammation and oxidative stress index

The levels of IL-6 and TNF-α were quantified using ELISA kits from the Nanjing Institute of Bioengineering, China. Meanwhile, the levels of Malondialdehyde (MDA) and Glutathione (GSH) were measured with ELISA kits supplied by Wuhan Saipei Biotechnology Co., Ltd., China. Following cessation of the color development, a microplate reader (BMG LABTECH, Offenburg, Germany) was employed to assess the OD at 450 nm.

### Dual-luciferase reporter assay

The wild-type (WT) or mutated (MUT) sequences of INO80 containing the binding sites of miR-642a-3p were designed by Gene Pharma (China) and inserted into the pGL3 luciferase vector (Promega, Madison, WI, USA). MC3T3-E1 cells were co-transfected with INO80-WT/MUT, miR-642a-3pM inhibitor/mimic, and the corresponding negative controls (NC) by Lipofectamine 2000 (Invitrogen, USA). Cell lysates were collected after 48 h of transfection, and the relative luciferase activity was quantified by normalizing firefly luminescence to Renilla luminescence with the dual-luciferase reporting kit (Promega, Shanghai, China).

### Western blot

Total protein was extracted by ice-cold RIPA lysis buffer (Beyotime, Shanghai, China). Concentration of protein was detected by the BCA Protein Quantification kit (YEASEN, Shanghai, China). Protein (30 µg) was isolated using 10% SDS-PAGE and subsequently transferred to a PVDF membrane (Invitrogen, Thermo Fisher Scientific, Inc.). The membranes were immunoblotted using specific primary antibodies (Rabbit anti-INO80: 18810-1-AP, 1: 1000, Proteintech, USA; Rabbit anti-GAPDH: AP0063, 1:5000, Bioworld Technology, USA) at 4 °C for 12 h, followed by secondary antibodies (HRP-conjugated Affinipure Goat Anti-Rabbit IgG (H + L), SA00001-2, 1: 5000, Proteintech, USA) at room temperature for 1 h. Images of protein bands were taken using the EasyBlot ECL kit (Sangon, Shanghai, China). The relative expression was normalized to GAPDH and then analyzed using ImageJ software 1.48u (National Institutes of Health, Bethesda, MD, USA).

### Statistical analysis

Data were analyzed using GraphPad Prism 10 and SPSS 23. The diagnostic performance of miR-642a-3p was evaluated using ROC analysis. The association between miR-642a-3p expression and BMD in patients was evaluated using the Pearson correlation analysis. Multivariate logistic regression assessed its predictive value for OPF. TargetScan was used to identify complementary binding sequences between miR-642a-3p and INO80. Two-group differences were compared by t-test, multi-group (≥ 3 groups) comparisons were performed via one-way ANOVA, and the cell proliferation was assessed via two-way ANOVA. All measurements were independently replicated three times, with three technical replicates included within each experiment to ensure data accuracy and reproducibility.

## Results

### Differential expression of miR-642a-3p and its diagnostic value

Clinical data from 91 HV and 127 PMO women with osteoporosis were evaluated. The PMO women were further categorized into OPF (*n* = 62) and OPNF (*n* = 65) subgroups. The differential expression of miR-642a-3p was evaluated by RT-qPCR. Results indicated no significant differences in age, BMI, Smoking and Drinking Habits, and History of hypertension and diabetes among the three groups (*P* > 0.05). This suggests the three groups were comparable. The three groups showed significant differences in all other clinical indicators, including Lumber spine bone mineral density (LS BMD), femur neck bone mineral density (FN BMD), total hip bone mineral density (TH BMD), Bone metabolic markers β-isomerized C-terminal telopeptides (β-CTx) and osteocalcin (OCN), E2, calcium (Ca), and 25-hydroxyvitamin D (25(OH)D) (*P <* 0.001, Table 1).

**Table 1:** Baseline information comparison between the HV and PMO groups

Indicators	HV group (*n* = 91)	PMO group (*n* = 127)		*P* value
		OPNF(65) OPF(62)		
Age	60.76 ± 6.61	60.98 ± 7.41	62.55 ± 5.59	0.228
BMI	23.99 ± 1.12	24.08 ± 1.51	24.30 ± 1.59	0.382
Smoking(No/Yes)	70/21	56/9	48/14	0.314
Alcohol intake(No/Yes)	65/26	52/13	45/17	0.450
Combined hypertension: (No/Yes)	54/37	47/18	46/16	0.095
Combined diabetes: (No/Yes)	52/39	43/22	42/20	0.331
LS BMD (g/cm^2^)	0.90 ± 0.07	0.72 ± 0.05	0.68 ± 0.05	<0.001
FN BMD (g/cm^2^)	0.80 ± 0.09	0.74 ± 0.04	0.70 ± 0.04	<0.001
TH BMD (g/cm^2^)	0.81 ± 0.09	0.70 ± 0.07	0.67 ± 0.06	<0.001
25(OH)D (ng/mL)	24.37 ± 3.44	23.24 ± 3.45	21.73 ± 2.05	<0.001
Ca (mmol/L)	2.39 ± 0.23	2.32 ± 0.14	2.22 ± 0.16	<0.001
β-CTx (ng/mL)	0.36 ± 0.04	0.56 ± 0.07	0.58 ± 0.06	<0.001
OCN (ng/mL)	8.02 ± 0.78	16.96 ± 1.89	18.22 ± 2.12	<0.001
E2 (pmol/L)	65.22 ± 8.95	30.17 ± 3.43	28.18 ± 3.11	<0.001

*BMI* Body Mass Index, *LS BMD* Lumber spine bone mineral density, *FN BMD* Femur neck bone mineral density, *TH BMD* Total hip bone mineral density, *25(OH)D* 25-hydroxyvitamin D, *Ca* Calcium, *β*-*CTx* β-isomerized C-terminal telopeptides, *OCN* Osteocalcin, *E2* Estradiol

**Fig. 1:**
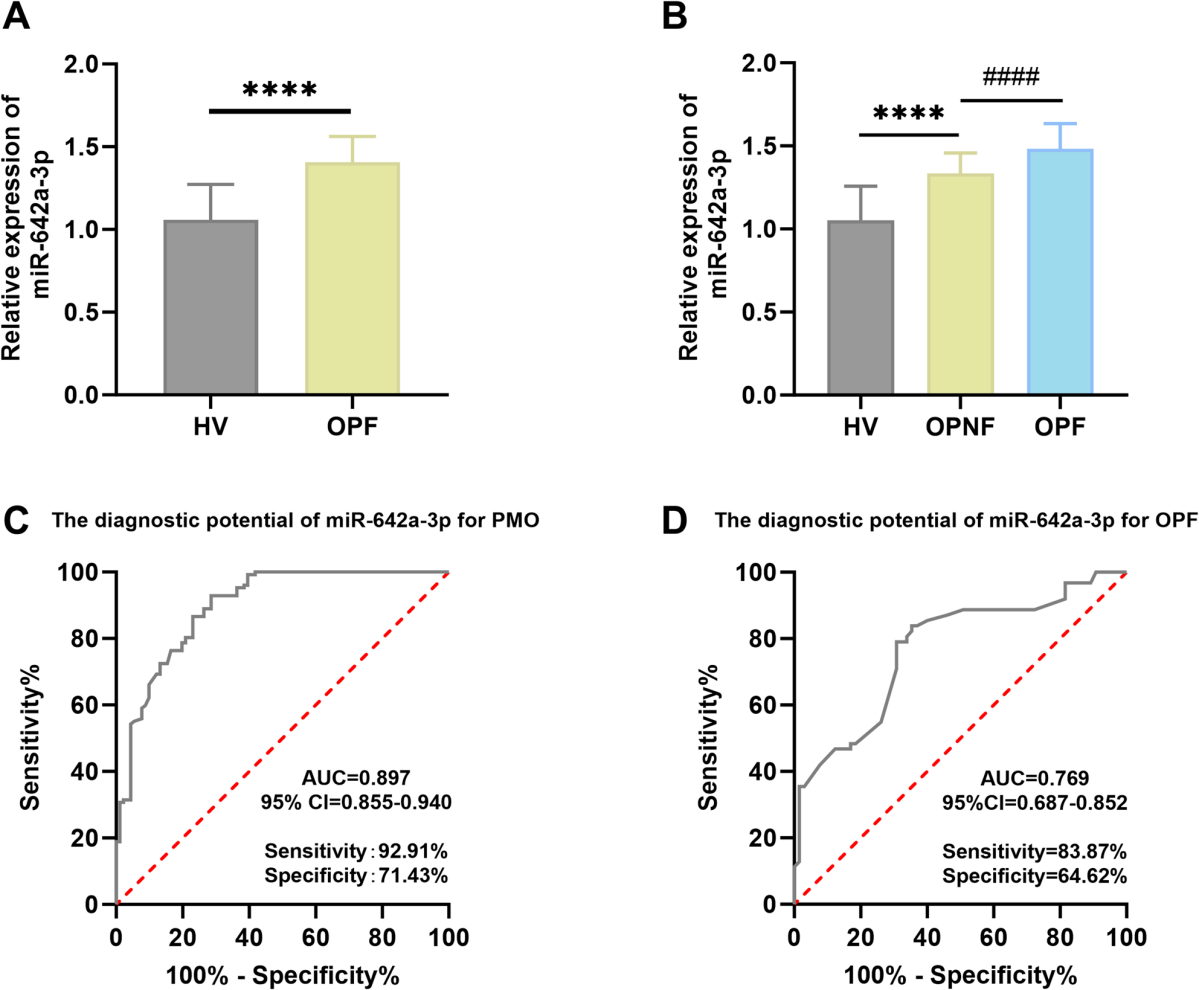
Differential expression of miR-642a-3p in PMO and ROC curves. **A** Relative expression of miR-642a-3p in 91 HV and 127 patients with PMO by RT-qPCR. **B** Differential expression of miR-642a-3p in 62 OPF compared to 65 OPNF by RT-qPCR. **C** ROC curve analysis evaluating the diagnostic potential of miR-642a-3p for PMO (sensitivity: 92.91%, specificity: 71.43%, AUC = 0.897, 95% CI: 0.855–0.940). **D** Plasma miR-642a-3p can effectively differentiate OPF patients from OPNF via ROC curve (sensitivity: 83.87%, specificity: 64.62%, AUC = 0.769, 95% CI: 0.687–0.852). (****P < 0.0001 vs. HV; ^####^*P* < 0.0001 vs. OPNF)

To evaluate the diagnostic potential of miR-642a-3p for PMO and OPF, ROC analysis was performed. MiR-642a-3p expression was markedly upregulated in PMO compared with HV (*P* < 0.0001, Fig. 1A), and its expression is markedly higher in OPF compared to the OPNF group (*P* < 0.0001, Fig. 1B). MiR-642a-3p can distinguish PMO patients from HV with high accuracy (AUC = 0.897, 95% CI: 0.855–0.940), the sensitivity and specificity were 92.91% and 71.43%, respectively (Fig. 1C). Moreover, as shown in Fig. 1D, miR-642a-3p also showed significant predictive value in distinguishing OPF from OPNF (sensitivity: 83.87%, specificity: 64.62%, AUC = 0.769, 95% CI: 0.687–0.852). In summary, miR-642a-3p is significantly upregulated in both PMO and OPF and shows strong diagnostic performance.

### Correlation between miR-642a-3p and BMD in patients

**Fig. 2:**
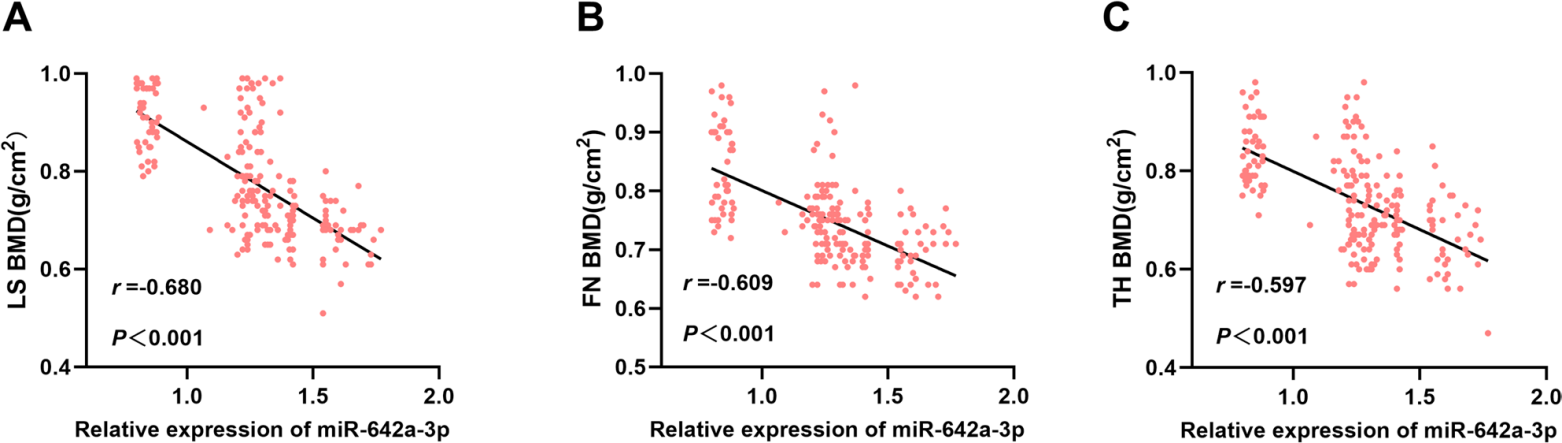
Correlation between miR-642a-3p and BMD in PMO patients. The expression of miR-642a-3p was negatively correlated with LS BMD (**A**, *r*=−0.680, *P* < 0.001), FN BMD (**B**, *r*=−0.609, *P* < 0.001), and TH BMD (**C**, *r*=−0.597, *P* < 0.001) using the Pearson correlation analysis

In order to confirm the correlation between miR-642a-3p and BMD in patients, we performed a Pearson correlation analysis. MiR-642a-3p was negatively correlated with LS BMD (Fig. 2A, *r*=−0.680, *P <* 0.001), FN BMD (Fig. 2B, *r*=−0.609, *P <* 0.001), and TH BMD (Fig. 2C, *r*=−0.597, *P <* 0.001) in patients with PMO. The lumbar spine is rich in cancellous bone. This strong correlation suggests that miR-642a-3p may be deeply involved in the pathological process of high metabolic rate bone loss, offering potential as a biomarker for monitoring early spinal bone loss. The femoral neck is the region with the highest risk of fracture. Its significant negative correlation with miR-642a-3p indicates that miR-642a-3p may assist in stratifying the risk of hip fractures. As a comprehensive indicator of total hip bone mass, the negative correlation between TH BMD and miR-642a-3p suggests that miR-642a-3p reflects not only localized changes in specific anatomical sites but also systemic imbalances in bone metabolism. Taken together, miR-642a-3p is negatively correlated with BMD across multiple skeletal sites, supporting its association with bone loss and fracture risk.

### Factors influencing OPF

To assess the factors influencing OPF, the logistic regression analysis was conducted. The differences in clinical indicators between the OPNF and OPF groups were analyzed using univariate logistic regression. Then, a multivariate logistic regression model was employed, with fracture status in PMO patients as the dependent variable (OPNF: 0, OPF: 1), and variables showing significant differences in univariate logistic regression as independent variables. Results indicated that after adjusting for other confounding factors, miR-642a-3p, β-CTx, and OCN were independent risk factors for the incidence of fractures in PMO patients. And LS BMD, FN BMD, and Ca were independent protective factors for OPF (Table 2).

**Table 2:** Logistic regression analysis of risk factors affecting osteoporotic fracture

**Parameters**	**Univariate analysis**			**Multivariate analysis**		
	***P***	**OR**	**95% CI**	***P***	**OR**	**95% CI**
Age	0.396	1.357	0.671–2.766			
BMI	0.179	1.615	0.804–3.281			
Smoking	0.200	1.815	0.731–4.709			
Alcohol intake	0.325	1.511	0.665–3.501			
Combined hypertension	0.810	0.908	0.411–1.996			
Combined diabetes	0.849	0.931	0.442–1.953			
LS BMD	0.003*	0.334	0.159–0.686	0.006*	0.223	0.073–0.626
FN BMD	<0.0001*	0.191	0.087–0.401	0.006*	0.238	0.081–0.641
TH BMD	0.038*	0.475	0.231–0.961	0.187	0.504	0.176–1.379
25(OH)D	0.022*	0.434	0.209–0.886	0.129	0.464	0.167–1.233
Ca	0.006*	0.367	0.176–0.746	0.008*	0.245	0.082–0.670
β-CTx	0.025*	2.241	1.107–4.614	0.011*	3.803	1.416–11.160
OCN	0.007*	2.730	1.317–5.817	0.043*	2.870	1.052–8.262
E2	<0.001*	0.257	0.118–0.541	0.080	0.407	0.145–1.105
miR-642a-3p	<0.0001*	6.469	3.039–14.400	0.003*	4.631	1.710–13.440

*BMI* Body Mass Index, *LS BMD* Lumber spine bone mineral density, *FN BMD* Femur neck bone mineral density, *TH BMD* Total hip bone mineral density, *25(OH)D* 25-hydroxyvitamin D, *Ca* Calcium, *β*-*CTx* β-isomerized, C-terminal telopeptides, *OCN* Osteocalcin, *E2* Estradiol. *P＜0.05

Collectively, these findings demonstrate that, beyond traditional bone metabolic markers and BMD measurements, miR-642a-3p serves as an independent risk predictor for OPF.

### Effects of miR-642a-3p on osteoblasts

**Fig. 3:**
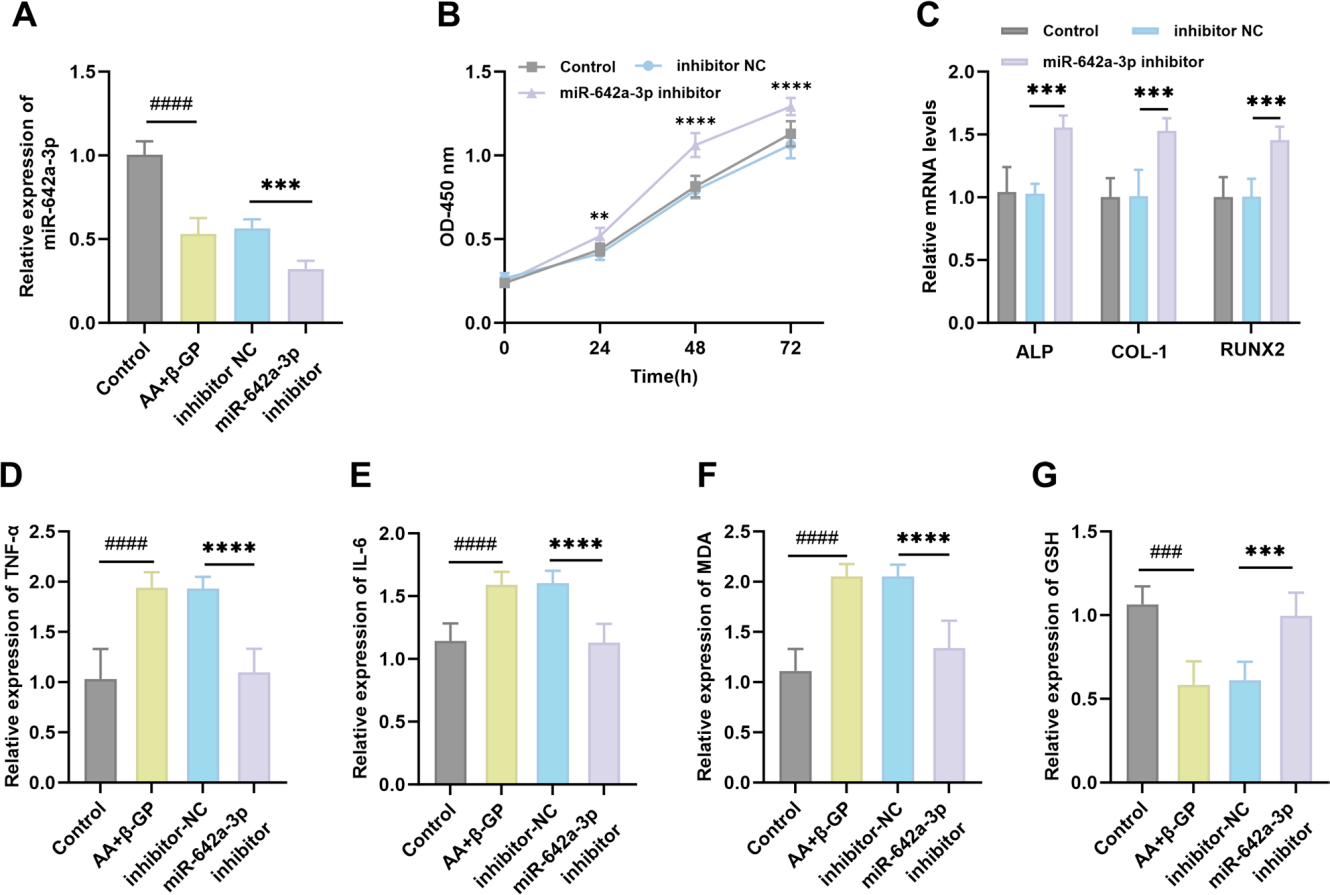
Effects of miR-642a-3p knockdown on osteoblast activity. **A** Following 15 days of osteoblast differentiation induction (using 50 µg/mL AA and 10 mM β-GP) and after transfection with 50 nM miR-642a-3p inhibitor for 48 h, the expression of miR-642a-3p was significantly downregulated. **B** MiR-642a-3p knockdown promotes proliferation of MC3T3-E1 cells by the CCK8 assay. **C** Silencing of miR-642a-3p led to elevated mRNA levels of osteoblast differentiation markers (ALP, COL-1, and RUNX2) by RT-qPCR. **D **and** E** Knockdown of miR-642a-3p markedly suppressed the expression levels of osteogenic differentiation-induced inflammatory mediators (IL-6 and TNF-α) using ELISA kits. **F **and** G** MDA levels were significantly elevated while GSH activity markedly decreased following osteogenic differentiation induction, whereas miR-642a-3p knockdown markedly restored the expression of these oxidative stress markers using ELISA kits. (*n* = 3 independent experiments, **P < 0.01, ***P < 0.001, ****P < 0.0001 vs. inhibitor NC; ^###^*P* < 0.001, ^####^*P* < 0.0001 vs. control)

To elucidate the cellular mechanisms by which miR-642a-3p influences bone metabolism, we conducted functional experiments in MC3T3-E1 osteoblasts. After 15 days of osteoblast differentiation, the expression of miR-642a-3p significantly decreased. And its expression was significantly reduced in MC3T3-E1 following transfection of miR-642a-3p inhibitor (Fig. 3A). MiR-642a-3p knockdown promoted proliferation in osteoblast MC3T3-E1 (Fig. 3B). The expression levels of osteogenic differentiation markers (ALP, COL-1, and RUNX2) were significantly elevated following miR-642a-3p knockdown (Fig. 3C). In addition, differentiated osteoblasts are accompanied by a certain degree of inflammation and oxidative stress. ELISA results revealed that miR-642a-3p knockdown markedly reduced the levels of inflammatory mediators TNF-α (Fig. 3D) and IL-6 (Fig. 3E) in differentiated osteoblasts. Following osteoblast differentiation induction, MDA levels significantly increased (Fig. 3F) while GSH activity markedly decreased (Fig. 3G) under LPS induction. However, miR-642a-3p knockdown significantly restored the expression of these oxidative stress markers. Thus, silencing miR-642a-3p exerts protective effects on osteoblasts by promoting differentiation and mitigating inflammatory and oxidative stress responses, highlighting its potential as a therapeutic target for osteoporosis.

### Interaction of miR-642a-3p with INO80

**Fig. 4:**
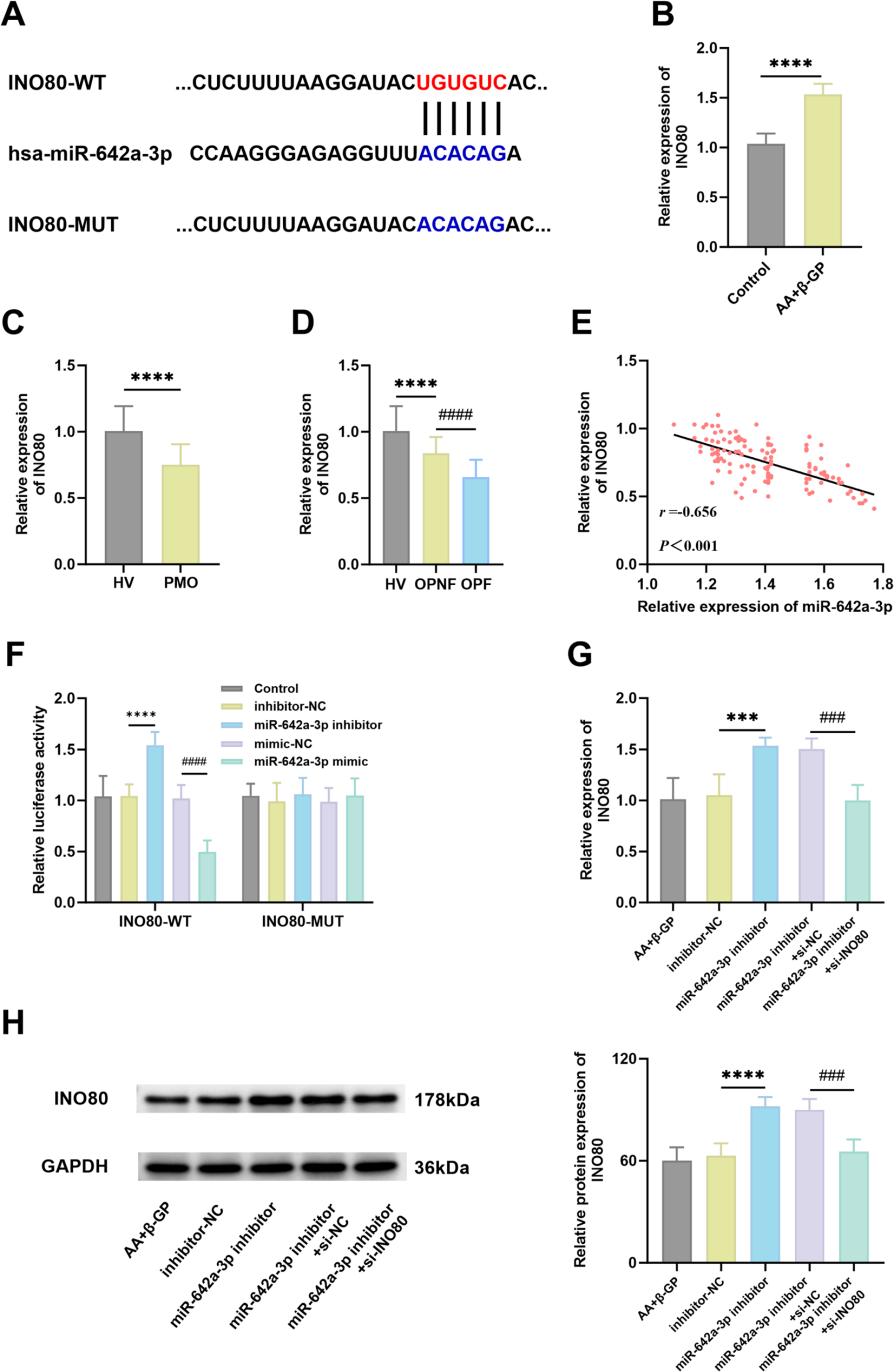
Interaction of miR-642a-3p with INO80. **A** INO80 was predicted as a miR-642a-3p target via the TargetScan database. **B** Expression of INO80 after 15 days of osteoblast differentiation induced by AA and β-GP by RT-qPCR. **C** Differential expression of INO80 in PMO compared to HV by RT-qPCR. **D** Relative expression of INO80 in OPF and OPNF patients by RT-qPCR. **E** INO80 expression is negatively linked to miR-642a-3p expression by the Pearson correlation analysis. **F** Dual-luciferase assay validated the interaction of INO80 with miR-642a-3p. **G** MiR-642a-3p inhibitor increased INO80 expression, and co-transfection of miR-642a-3p inhibitor and si-INO80 reversed the effect by RT-qPCR. **H** Western blot analysis revealed that INO80 protein levels in MC3T3-E1 cells. The miR-642a-3p inhibitor upregulated INO80 protein expression, while this effect is reversed by si-INO80. (*n* = 3 independent experiments, ***P < 0.001, ****P < 0.0001 vs. HV, si-NC or inhibitor NC; ^###^*P* < 0.001, ^####^*P* < 0.0001 vs. OPNF, oe-NC or miR-642a-3p inhibitor + si-NC)

The interaction between miR-642a-3p and INO80 was predicted using the database and validated by a dual-luciferase assay. The binding sites between miR-642a-3p and INO80 were identified using the TargetScan database (Fig. 4A). After 15 days of osteoblast differentiation, INO80 expression was found to be upregulated (Fig. 4B). INO80 exhibits significantly reduced expression in patients with PMO (Fig. 4C), while its expression is markedly lower in OPNF patients (Fig. 4D). MiR-642a-3p expression is negatively related to INO80 expression (*r* = − 0.656, *P* < 0.001, Fig. 4E). Silencing miR-642a-3p markedly promote INO80 luciferase activity, while the miR-642a-3p overexpression leads to a significant decrease. However, following a mutation in INO80, the luciferase activity was not affected (Fig. 4F). MiR-642a-3p knockdown increased INO80 expression, and co-transfection of miR-642a-3p inhibitor and si-INO80 reversed the effect (Fig. 4G). Furthermore, western blot analysis revealed that the miR-642a-3p inhibitor markedly increased INO80 protein expression in MC3T3-E1 cells; this effect was abrogated by si-INO80 (Fig. 4H). Overall, miR-642a-3p directly targets and regulates INO80, and these molecules may serve as important therapeutic targets for the diagnosis of PMO and the prediction of OPF risk.

## Discussion

In this study, miR-642a-3p may hold potential as both a diagnostic biomarker for early-stage PMO and a prognostic indicator for OPF risk. MiR-642a-3p is significantly negatively correlated with LS BMD, FN BMD, and TH BMD. MiR-642a-3p knockdown promotes osteoblast proliferation, inflammation, and oxidative stress; the possible regulatory mechanism is through targeting INO80.

MiR-642a-3p expression was found to be significantly upregulated in PMO. Its expression was significantly upregulated in OPF patients compared to OPNF patients, highlighting its critical role in the development of osteoporosis to fracture. And the ROC curve analysis indicates that miR-642a-3p holds great promise for the early diagnosis of PMO and the assessment of OPF risk. Similarly, extensive research indicates that miRNAs can influence the progression of PMO and OPF by affecting both bone formation and bone resorption. For example, Sun et al. reported that miR-595 might be a risk factor for decreased bone mass in postmenopausal women with hip fractures [22]. Qian et al. pointed out that serum miR-208a-3p served as a diagnostic biomarker for PMO and a risk factor for OPF [23].

MiR-642a-3p exhibits a significant negative correlation with LS BMD, FN BMD, and TH BMD, which partially explains its important role in the diagnosis and treatment assessment of PMO and OPF. LS BMD correlates with the most common vertebral fractures, while FN BMD and TH BMD are crucial for assessing the risk of the most dangerous hip fractures. The combination of BMD and TBS that reflects bone structure degeneration can enhance the clinical assessment of fracture risk [24]. Multivariate logistic regression indicated that upregulation of miR-642a-3p, OCN, and β-CTx were independent risk factors for OPF, while LS BMD, FN BMD, and Ca intake were independent protective factors. OCN secreted by osteoblasts is a key marker of bone formation, while β-CTx, a degradation product of type I collagen released during osteoclast activity, is a biomarker of bone resorption and is crucial for maintaining skeletal integrity [25, 26]. Ca deficiency increases bone resorption and fracture risk in postmenopausal women, closely affecting bone health [27]. These results indicate that miR-642a-3p may have potential as a biomarker for future clinical prediction of PMO and OPF, building upon existing clinical assessment factors.

Osteoblast dysfunction plays a pivotal role in the pathogenesis of osteoporosis and fractures. Therefore, investigating the function of miR-642a-3p in osteoblasts is crucial for unraveling the molecular mechanisms of osteoporosis. This study found that miR-642a-3p knockdown promotes osteoblast proliferation, thereby inhibiting the progression of osteoporosis and fractures. And miR-642a-3p inhibition led to elevated expression of ALP, COL-1, and RUNX2 in induced osteoblasts. ALP, COL-1, and RUMX2 are common osteogenesis-related genes that promote bone formation through different pathways [28]. Therefore, miR-642a-3p may influence the progression of osteoporosis and fractures by regulating genes associated with osteoblastic differentiation. Osteoblasts following induced differentiation exhibit a certain degree of inflammatory and oxidative stress responses. The findings of this study indicate that miR-642a-3p knockdown alleviates inflammatory responses and oxidative damage in osteoblasts following induced differentiation, thereby reducing the risk of OPF. Research confirms that the pathogenesis of PMO is closely associated with immune dysfunction and systemic inflammatory activation. Following menopause, the decline in E2 removes a protective shield, resulting in a progressive buildup of various inflammatory cytokines, such as TNF-α and IL-6 [29]. Furthermore, these inflammatory cytokines mediate oxidative stress damage, inhibit osteoblast mineralization, and disrupt the dynamic equilibrium between bone resorption and formation, progressively contributing to osteoporosis and related fractures [30]. MDA serves as a biomarker for assessing oxidative stress-induced cellular damage, while GSH protects cells from oxidative injury by scavenging free radicals [31]. Consequently, when osteoblasts undergo oxidative stress, MDA levels increase while GSH activity decreases.

Deeper understanding of miRNA–mRNA regulatory interactions could offer new avenues for developing novel diagnostic markers and treatment strategies for PMO and OPF [32]. Research indicates that the interaction between miR-370-3p influences the development of PMO by targeting INO80 [33]. This study revealed significantly reduced INO80 expression in patients with osteoporotic fractures, which showed a significant negative correlation with miR-642a-3p expression. MiR-642a-3p can directly bind to and regulate INO80. Therefore, the potential mechanism by which miR-642a-3p promotes osteoporosis and fracture progression may be achieved through regulating the INO80 protein expression. The combined effects of these two factors on osteoblasts warrant further investigation.

However, this study has other limitations. PMO is considered to result from insufficient E2. Further research is needed to evaluate the effects of E2 on miR-642a-3p expression and bone metabolism. And owing to the complex regulatory mechanisms of miRNAs, subsequent studies will explore miRNA functional patterns and detailed signaling pathways in depth by further animal and molecular experiments.

This study employed MC3T3-E1 cells, which exhibit rapid proliferation, stable characteristics, and ease of cultivation, making them a highly representative in vitro model for bone metabolism research. Utilizing this cell line allows for the direct regulatory role of miR-642a-3p in the osteogenic pathway to be focused upon, whilst simultaneously eliminating donor-specific variability. However, MC3T3-E1 cells are derived from mice and constitute an immortalized cell line, whose metabolic and regulatory pathways may differ from those of human primary osteoblasts. Consequently, the conclusions of this study carry certain limitations when directly translated to clinical applications. In the future, we shall conduct further validation in human-derived cell models (such as hBMSCs or hFOB1.19) to enhance the clinical translational relevance of our findings. Moreover, this study only assessed the transcriptional level of INO80 in clinical samples using RT-qPCR, without further validation at the protein level using Western blot. This limitation restricts the ability to directly infer the biological effects of INO80 at the level of protein function. Exosomes, as actively secreted and protected functional vectors from living cells, offer a window into plasma for achieving more precise, dynamic, and actionable disease characterization. We will use a kit to extract RNA from the exosomes and then comprehensively characterize the size distribution, morphology, and exosomal markers (CD9, CD63, CD81, and calnexin) of the purified exosomes to rule out any potential interference from non-exosomal vesicles or macromolecular complexes on the test results.

Overall, the study indicated that miR-642a-3p is a promising marker for the diagnosis of PMO and OPF. High miR-642a-3p expression is associated with increased fracture risk. MiR-642a-3p knockdown can promote osteoblast proliferation, alleviate inflammatory responses and oxidative damage, as well as regulate osteoblast differentiation by modulating relevant osteogenic genes. The underlying mechanism of these effects may be related to targeting INO80. 

## Data Availability

The datasets used and/or analysed during the current study are available from the corresponding author on reasonable request.
